# Predator-by-Environment Interactions Mediate Bacterial Competition in the *Dictyostelium discoideum* Microbiome

**DOI:** 10.3389/fmicb.2018.00781

**Published:** 2018-04-24

**Authors:** R. Fredrik Inglis, Odion Asikhia, Erica Ryu, David C. Queller, Joan E. Strassmann

**Affiliations:** Department of Biology, Washington University in St. Louis, St. Louis, MO, United States

**Keywords:** siderophores, *Pseudomonas fluorescens*, Gac regulatory system, cooperation, predation

## Abstract

Interactions between species and their environment play a key role in the evolution of diverse communities, and numerous studies have emphasized that interactions among microbes and among trophic levels play an important role in maintaining microbial diversity and ecosystem functioning. In this study, we investigate how two of these types of interactions, public goods cooperation through the production of iron scavenging siderophores and predation by the social amoeba *Dictyostelium discoideum*, mediate competition between two strains of *Pseudomonas fluorescens* that were co-isolated from *D. discoideum*. We find that although we are able to generally predict the competitive outcomes between strains based on the presence and absence of either *D. discoideum* or iron, predator-by-environment interactions result in unexpected competitive outcomes. This suggests that while both cooperation and predation can mediate the competitive abilities and potentially the coexistence of these strains, predicting how combinations of different environments affect even the relatively simple microbiome of *D. discoideum* remains challenging.

## Introduction

Interactions within and between microbial species play an important role in mediating competition and shaping their evolutionary histories. These types of interactions range from producing antimicrobial compounds that kill potential competitors to cooperatively scavenging poorly accessible nutrients such as iron from the environment and have been implicated in a number of important evolutionary processes such as generating and maintaining diverse microbial communities (Kerr et al., 2002; Kirkup and Riley, 2004; Cordero et al., 2012a,b; Biernaskie et al., 2013; Coyte et al., 2015; Inglis et al., 2016; Leinweber et al., 2017).

One key mechanism that has been shown to influence microbial interactions and competition between species is predation (Meyer and Kassen, 2007; Jousset et al., 2009; Ishii and Shimada, 2012; Friman et al., 2013). Predation is able to increase microbial diversity through adaptive radiations caused by frequency dependent selection where predators evolve to be best at hunting and eating the most common type (Meyer and Kassen, 2007). Conversely, predation can also be important in preventing the spread of new genetic mutants such as microbial cheaters, if the cheaters that no longer produce anti-predator toxins are preferentially eaten (Jousset et al., 2009; Friman et al., 2013). This suggests that predation can have a variety of effects on both interacting microbes and complex microbial communities. Interactions between trophic levels may therefore play a key role in determining the species composition of diverse populations of interacting microbes.

Complex trophic interactions have previously been illustrated in the social amoeba *Dictyostelium discoideum*, which eats a variety of soil bacteria and forms symbiotic relationships with a number of different bacterial species (Brock et al., 2011; DiSalvo et al., 2015). *D. discoideum* is found on the forest floor, where it inhabits the soil and leaf litter. *D. discoideum*’s primary food source is bacteria which it predates on during its vegetative growth stage as single celled amoebae. During this feeding stage *D. discoideum* comes into contact with a number of different soil associated bacteria and has been shown to form symbiotic relationships with a number of different *Burkholderia* species and *Pseudomonas fluorescens* (Brock et al., 2013; Stallforth et al., 2013; DiSalvo et al., 2015). In these symbioses, the amoebas disperse the bacteria to new locations, while the bacteria provide food or other services to the amoebas (Brock et al., 2011, 2013, 2016a,b; Stallforth et al., 2013; DiSalvo et al., 2015). These symbioses are thought to be mostly driven by the presence of certain *Burkholderia* (DiSalvo et al., 2015), but these *Burkholderia* allow *D. discoideum* to also carry other bacterial species such as *P. fluorescens* that can act as important sources of food and produce beneficial compounds for *D. discoideum* (Stallforth et al., 2013). For example, *P. fluorescens* strains PfA-QS161 (Pf2) and PfB-QS161 (Pf3) have both been cultured from the same clonal isolate of *D. discoideum* (Stallforth et al., 2013; **Table 1**). Pf3 acts as a food source and Pf2, though inedible, produces small molecules beneficial to the amoebae (Stallforth et al., 2013). Interestingly, previous work suggests Pf3 differs in its edibility and chemical profile due to a single mutation in the GacS/GacA two-component regulatory system and phylogenetic analysis shows that it is likely to be derived from Pf2 (Stallforth et al., 2013; **Table 1**). However, these two strains differ by at least 135 other SNPs (Stallforth et al., 2013), suggesting that this association has continued for some time, even though both strains can also grow independently.

**Table 1:** An outline of the experimental conditions (i.e., with our without iron, with or without *D. discoideum*, and with or without direct interactions between strains), the predicted strain that should win the competition, the actual outcome, and a brief explanation of the results.

Grown Separately			Grown Together		
	+ Iron	- Iron		+ Iron	- Iron
+ *D. discoideum*	Prediction: Pf2	Prediction: ?	+ *D. discoideum*	Prediction: Pf2	Prediction: Pf2
	Outcome: Pf2	Outcome: Pf3		Outcome: Pf2	Outcome: Pf3
	Reason: *D. discoideum* eats Pf3.	Reason: Poor *D. discoideum* growth under these conditions.		Reason: *D. discoideum* eats Pf3.	Reason: Poor *D. discoideum* growth under these conditions.
-*D. discoideum*	Prediction: ?	Prediction: Pf3	-*D. discoideum*	Prediction: ?	Prediction: Pf2
	Outcome: Pf3	Outcome: Pf3		Outcome: Pf3	Outcome: Pf2
	Reason: Increased growth presumably due to *gacA* mutation as observed in Pf2Δ*gacA*.	Reason: Increased pyochelin production in Pf3.		Reason: Increased growth presumably due to *gacA* mutation as observed in Pf2Δ*gacA*.	Reason: Pf2 utilizes the pyochelin produced by Pf3.

GacS/GacA mutants are fairly common in the environment (Jousset et al., 2009; Driscoll et al., 2011) and have a disrupted environmental sensing system, which can lead gene expression changes for up to 10% of the genome (Hassan et al., 2010). The loss of GacS/GacA function often involves regulatory changes to extra-cellular products and loss of secondary metabolism (van den Broek et al., 2005). This is accompanied by an increase in growth compared the wild-type strain, which may allow better colonization of new environmental niches (van den Broek et al., 2005). In Pf3 this loss of function in GacS/GacA system results in the increased expression of pyochelin, an iron-chelating siderophore that acts as a cooperative public good (Dumas et al., 2013; Stallforth et al., 2013). From an evolutionary perspective this is interesting, because selection appears to have favored a macro-mutation that affects a great many traits. However, from an ecological standpoint some of these changes may provide the niche differences that may facilitate coexistence between these strains.

We therefore sought to test whether environmental conditions pertinent to the difference in GacS/GacA expression, such as presence or absence of iron and predation by *D. discoideum*, can explain the competitive abilities of these two strains. Taking these environmental factors singly, we can make the following predictions (see also **Table 1**):

1a. Presence of *D. discoideum* should favor Pf2, because Pf3 is edible (Stallforth et al., 2013).
1b. Absence of *D. discoideum* should favor Pf3, assuming predator defense is costly (Steiner, 2007; Jousset et al., 2010).
2a. Iron-rich medium should favor Pf2, assuming the production of siderophores by Pf3 is costly (Ross-Gillespie et al., 2007; Kummerli and Brown, 2010).
2b. Iron-poor medium should favor siderophore-producing Pf3 when the two clones are grown separately, but when they are grown mixed together, Pf2 should do better by gaining the benefit of Pf3’s siderophores without paying the cost (Griffin et al., 2004).

**FIGURE 1:**
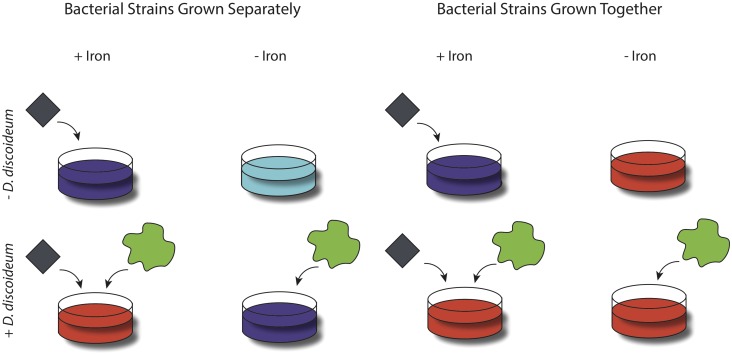
A cartoon schematic of the experimental design. Bacterial strains were grown either separately or together in iron-rich and iron limited media (as represented by the gray diamond), in both the presence and absence of *D. discoideum* (as represented by the green amoeba). Colors of the petri dish refer to which strain we predict to win the competition between strains based on their competitive abilities as outlined in **Table 1**. If the petri dishes are colored red we predict Pf2 to win and if colored blue we predict Pf3 to win. When there is no clear prediction petri dishes are colored purple.

We tested these predictions in a fully factorial design varying (1) presence or absence of *D. discoideum* (2) iron-rich or iron-poor media and (3) whether the *P. fluorescens* strains are grown separately or mixed together. **Figure 1** shows the design. Where both factors predict that one the two clones should do better (e.g., *D. discoideum* + and Iron + both favor Pf2) then we predict that clone will outcompete the other (shown in **Figure 1** as red for Pf2 or blue for Pf3). When one factor favors one clone and the other factor favors the other clone (such as *D. discoideum* - and Iron +), we make no prediction (shown as purple) but the outcomes might show interesting interactions. A final prediction concerns the genetic basis of these competitive abilities. If the GacS/GacA regulatory system is responsible then a mutant of Pf2 with a defective in GacS/GacA would act similarly to Pf3 in all environments.

## Materials and Methods

### Strains and Culture Conditions

In order to investigate how predation affects bacterial social interactions we used a naturally isolated *D. discoideum* QS161 which we isolated from forest soil at Mountain Lake Biological Station and two strains of the bacterium *P. fluorescens* Pf2 (previously referred to as PfA) and Pf3 (previously referred to as PfB) isolated from QS161, as described in Stallforth et al. (2013) (**Table 2**). The chemical profiles and edibility of Pf2 and Pf3 can presumably be attributed a single stop-codon in the GacS/GacA regulatory system of Pf3, which causes the loss of a highly conserved DNA binding domain of the *gacA* gene. We therefore also made use of a previously constructed Pf2 strain with a deletion of the *gacA* gene (Pf2Δ*gacA*) (Stallforth et al., 2013) to test whether competitive differences between Pf2 and Pf3 could be explained by this mutation alone. In order to distinguish between these strains during competition assays, we tagged both Pf3 and Pf2 Δ*gacA* with a chromosomal insertion of YFP (*att*Tn7::*e-yfp*) which allowed us to distinguish these strains when competed against an untagged Pf2, and used these strains for all subsequent experiments (Choi and Schweizer, 2006). We cured QS161 cured of associated bacterial symbionts by culturing with tetracycline (DiSalvo et al., 2015).

**Table 2:** Description of the strains used in this study and how they vary.

	Pf2	Pf2Δ*gacA*	Pf3
GacS/GacA	Functional	Defective	Defective
Siderophore production	Functional	Increased	Increased
Food source for *D. discoideum*	No	Yes	Yes
Secretion of small molecules beneficial of *D. discoideum*	Yes	No	No

We revived *D. discoideum* QS161 from frozen glycerol stocks by re-suspending spores in 100ul KK2 [2.25 g KH_2_PO_4_ (Sigma-Aldrich) and 0.67 g K_2_HPO_4_ (Fisher Scientific) per liter] and plating on SM/5 agar plates [2 g glucose (Fisher Scientific), 2 g Bacto Peptone (Oxoid), 2 g yeast extract (Oxoid), 0.2 g MgCl_2_ (Fisher Scientific), 1.9 g KHPO_4_ (Sigma-Aldrich), 1 g K_2_HPO_5_ (Fisher Scientific), and 15 g agar (Fisher Scientific) per liter] with 100 ul of *Klebsiella pneumoniae*, diluted to an OD_600nm_ of 1.5, and incubating at room temperature. All three strains of *P. fluorescens* (Pf2, Pf3, and Pf2 Δ*gacA*) were grown from frozen glycerol stocks in LB broth (Fisher Scientific) shaking at 220 rpm at room temperature.

### Measuring Iron-Chelating Ability

Previous work has identified that *P. fluorescens* strains Pf2 and Pf3 differ in their production of the iron-chelating siderophore, pyochelin. We therefore, first tested whether the production of pyochelin in Pf3 results in an increased ability to chelate iron from its environment (Supplementary Figure S1). We measured each strain’s relative ability to chelate iron by growing six replicate populations (Pf2, Pf3, and Pf2 Δ*gacA*) in iron limited CAA media [5 g casamino acids, 1.18 g K_2_HPO_4_^∗^ 3H_2_O (Sigma-Aldrich), 0.25 g MgSO_4_^∗^ 7H_2_O (Sigma-Aldrich) per liter supplemented with 20 mM NaHCO_3_ (Sigma-Aldrich) and 100 μg/ml human apo-transferrin (Sigma-Aldrich)] shaking at room temperature for 24 h. We then performed a modified chrome azurol S (CAS) assay in order to measure the overall iron-chelating ability of each strain (Schwyn and Neilands, 1987; Inglis et al., 2016). We added 100 μl of CAS solution to 100 μl of bacterial culture. We incubated this mixture at room temperature, in darkness for 30 min, and we measured the color change associated with the removal of iron (through the activity of siderophores) from the CAS solution using absorbance at 630 nm (Infinite 200 PRO, Tecan, Switzerland). We also measured bacterial density using OD at 600 nm in the samples before the CAS solution had been added, and we calculated iron-chelating activity as [1 - (*A_i_*/*A*_ref_)]/(Density*_i_*), where *A* = absorbance at 630 nm of the assay mixture (Supplementary Figure S1).

### Competition Experiments

We performed competition experiments between our three bacterial strains (Pf2, Pf3, and Pf2 Δ*gacA*) (**Figure 1**). These strains were grown both separately and mixed together in direct competition, in both presence and absence of *D. discoideum*, and in two different environments: relatively iron rich and iron poor. In order to perform these competitions we harvested *D. discoideum* amoebae after 40 h of growth and re-suspended them in KK2 with a density of 10^7^ amoebas per milliliter. We grew overnight cultures of Pf2, Pf3, and Pf2Δ*gacA* and diluted each to an OD_600nm_ of 1. When strains were grown in the presence of *D. discoideum*, we plated 100 ul of the amoeba suspension with 100 ul of either a single strain or 50 ul of both competing strains (making a total of 100 ul). When strains were grown in the absence of *D. discoideum*, we substituted the addition of amoebae with 100 ul KK2. We plated these various combinations of *P. fluorescens* and *D. discoideum* on CAA agar supplemented with either 100 uM FeCl_3_ (for relatively iron rich environments) or with 20 mM NaHCO_3_ (sodium bicarbonate) and 100 μg/ml human apo-transferrin (for relatively iron poor environments), with six replicates for each unique combination. These combinations include the presence and of *D. discoideum*, in iron rich and iron limited media, for all three strains growing separately and pairwise competitions between Pf2 vs. Pf3 and Pf2 vs. Pf2Δ*gacA*. In order to correct for effects that iron rich and iron poor media might have on *D. discoideum* growth, we grew amoebae in both conditions supplementing both with our standard laboratory food bacterium *K. pneumoniae*, finding no difference in growth between iron rich and iron poor media (Supplementary Figure S2).

We incubated the plates at room temperature for 96 h. We subsequently harvested both bacteria and amoebae by flooding individual plates with 5 ml of KK2 and then re-suspended each mixture in 15 ml falcon tube. We then performed a low gravity spin (3 min at 310 rcf) to separate the bacteria from the amoebae. We counted both amoebae and bacteria using flow cytometry (BD Accuri C6). We stained bacterial samples with SYTO-62 (Thermo Fisher) in order to obtain a total count of the bacterial population and the numbers of Pf3 and Pf2 Δ*gacA* could easily be differentiated because they express YFP. Additionally we added cell-counting beads (Thermo Fisher) to each sample to standardize counts between samples.

### Flow Cytometry and Statistical Analyses

Flow cytometry data were analyzed using Bioconductor (Gentleman et al., 2004) as previously described (Smith et al., 2016). We used a linear model to analyze our bacterial density data. We used the presence and absence of iron, the presence and absence of *D. discoideum*, strain identity, and whether strains were grown/competed together or separately as factors (fixed effects) to explain bacterial density (bacterial density ∼ iron^∗^strain^∗^*D. discoideum*^∗^competition). *T*-tests were used to compare between strains in the different conditions and listed in **Table 3**. All statistics were performed in R version 3.2.3 (R Core Team, 2015).

**Table 3:** Summary statistics for the main model effects (iron, *D. discoideum*, Strain, and Competition) and relevant strain comparisons.

	Degrees of freedom	*F*-value	*t*-Value	*p*-Value
Iron	12	11.10	–	1.8E-15
*D. discoideum*	12	9.71	–	1.123E-13
Strain	16	9.80	–	3.026E-16
Competition	12	9.13	–	6.71E-13
Pf2 vs. Pf2Δ*gacA*	106	–	-5.62	1.63E-05
Pf3 vs. Pf2Δ*gacA*	70	–	-1.18	0.24
Pf3 vs. Pf2 (iron, *D. discoideum*, separate)	10	–	6.68	0.0002
Pf3 vs. Pf2 (iron, *D. discoideum*, together)	10	–	4.62	0.0007
Pf3 vs. Pf2 (iron, no *D. discoideum*, separate)	10	–	-4.82	0.0004
Pf3 vs. Pf2 (iron, no *D. discoideum*, together)	10	–	-2.21	0.049
Pf3 vs. Pf2 (no iron, *D. discoideum*, separate)	10	–	-8.2	3E-06
Pf3 vs. Pf2 (no iron, *D. discoideum*, together)	10	–	-4.88	0.0005
Pf3 vs. Pf2 (no iron, no *D. discoideum*, separate)	10	–	-8.2	3.06E-06
Pf3 vs. Pf2 (no iron, no *D. discoideum*, together)	10	–	13.36	3.8E-05

*Strain comparisons include Pf2 and Pf3 compared to Pf2ΔgacA across the whole data-set and specific strain comparisons for each condition. These strain comparisons combined the data from Pf3 and Pf2ΔgacA, as there was no significant difference between these strains, and were compared to Pf2. Comparisons were either unpaired or paired *t*-tests depending on whether the strains where grown separately (unpaired) or together (paired).*

## Results

**FIGURE 2:**
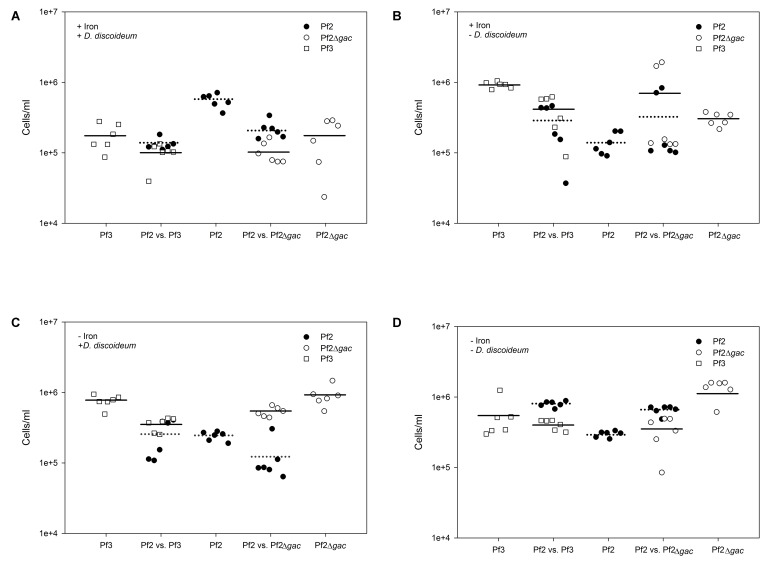
Bacterial densities across all competitions in the presence and absence of both iron and *D. discoideum*. Bacterial densities in the strains grown separately (i.e., single strains) were divided by 2 to give a per capita measure of cells produced. Horizontal lines represent the mean bacterial density of the strain. Dotted lines represent the mean of Pf2, whereas solid lines represent the means of Pf3 or Pf2Δ*gacA*. **(A)** In the presence of *D. discoideum* and iron-rich media, Pf2 outcompetes Pf3 and Pf2Δ*gacA* both when it is grown separately and together. **(B)** However, in the absence of *D. discoideum* and in the presence of iron, Pf3 and Pf2Δ*gacA* outcompete Pf2. **(C)** In iron-limited environments when *D. discoideum* is present, this pattern is surprisingly maintained. **(D)** However, when *D. discoideum* is absent in iron-limited environment, Pf2 grows poorly on its own, and it is able to outcompete Pf3 and Pf2Δ*gacA* when grown together. This suggests it is acting like a social cheater and exploiting the pyochelin produced by Pf3 and Pf2Δ*gacA*.

Our results reveal a context-dependent pattern of competitive fitness between Pf2 and Pf3 that depend on two environmental conditions: the presence of iron and *D. discoideum* that support four out of our five initial predictions (summarized in **Table 1**). As predicted, we find that the Pf2Δ*gacA* mutant and Pf3 behave similarly across all our experimental treatments and reach similar bacterial densities (*t* = -1.18, *p* > 0.24) (**Figure 2** and **Table 3**). This suggests that although Pf2 and Pf3 differ by over 100 SNPs, their growth and competitive abilities under these experimental conditions can in large part be explained by a non-functional GacS/GacA regulatory system (Stallforth et al., 2013). This is perhaps somewhat unsurprising as the GacS/GacA regulatory system is responsible for regulating up to 10% of the *P. fluorescens* genome (Hassan et al., 2010), but represents an interesting example of a single mutation conferring large competitive fitness effects that vary considerably over different environments. However, it is also important to note that changes to the GacS/GacA system may affect more than just pyochelin production and edibility, so some of the competitive fitness differences we observe may be due to other functions of GacS/GacA.

**FIGURE 3:**
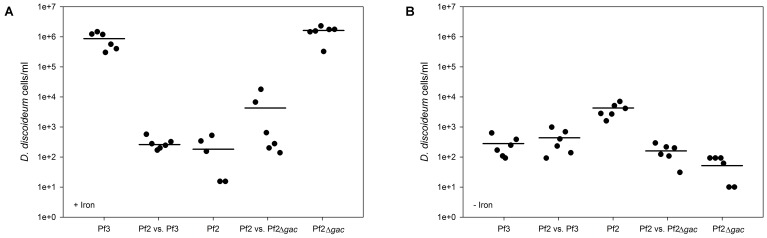
*D. discoideum* densities in iron-limited and iron-rich environments after 96 h. **(A)** Amoebae reach the highest densities in iron-rich environments, when grown in the presence of Pf3 and Pf2Δ*gacA.* **(B)** When grown in iron-limited environments *D. discoideum* has overall lower densities but has the highest density in the presence of Pf2.

We also find that when the strains are grown separately, we see a clear correspondence to our initial predictions. Pf2 grows better in iron-rich media in the presence of *D. discoideum* compared to Pf3 (*t* = 6.68, *p* < 0.002) (**Figures 2A**, **3A** and **Table 3**), probably because Pf3 is eaten by *D. discoideum* under these conditions and Pf2 presumably does not pay the cost of producing siderophores that are not needed (Ross-Gillespie et al., 2007; Kummerli and Brown, 2010). This contrasts to environmental conditions where iron is limiting and no *D. discoideum* is present as now Pf3 reaches higher densities compared to Pf2 (*t* = -8.2, *p* < 0.001) (**Figure 2D** and **Table 3**), presumably as Pf3 is able to obtain more iron from the environment through the overexpression of siderophores and because it also probably avoids Pf2’s costs of predator defense (Steiner, 2007; Jousset et al., 2010).

In the cases where each strain is expected to be favored by one of the two environmental variables, somewhat surprisingly we found that Pf3 outcompeted Pf2 in both these sets of conditions when grown separately (iron-rich with no *D. discoideum* (*t* = -4.82, *p* < 0.001) and iron-limited with *D. discoideum* (*t* = -8.2, *p* < 0.001) (**Figures 2B,C** and **Table 3**). Pf2Δ*gacA*’s ability to reach higher final densities in the iron-rich conditions where no *D. discoideum* is present shows that the GacS/GacA mutation confers a simple growth yield advantage under these conditions. In iron-limited environments with *D. discoideum* Pf3 should suffer from predation by the amoebae, but we again find that Pf3 reaches higher final densities which can likely be explained by a combination of the growth enhancing effects of over expressing siderophores and amoebae’s poor growth in the presence of Pf3 under these conditions (**Figure 3B** and **Table 1**), which reduces their predatory ability. This reduction in growth might be in part due to expression changes in Pf3 under these conditions, which affect amoebae growth.

When Pf2 and Pf2Δ*gacA* are grown together in iron-rich media without the presence of *D. discoideum*, conditions that can either favor both Pf2 or PfΔ*gacA*, we see that Pf2Δ*gacA* outcompetes Pf2 and reaches higher final densities (*t =* -2.21, *p* < 0.049) (**Figure 2B** and **Table 3**). This is the same result as when the strains are grown separately, again illustrating that the *gac* mutation confers some general growth yield advantage in iron-rich conditions. This increase in final density observed in Pf3 and the Pf2 *gacA* mutant (Pf2Δ*gacA*) may help explain the relatively frequent occurrence of *gac* mutants in nature (Driscoll et al., 2011), which may simply be due to direct fitness benefits in some environments through drastic expression changes.

When Pf2 and Pf3 are grown together in iron-rich environments in the presence of *D. discoideum* Pf2 reaches final higher densities because the amoebae predate on Pf3, as seen when grown separately (*t* = 4.62, *p* < 0.001) (**Figures 2A**, **3A** and **Table 3**). However, under iron-limited conditions where no *D. discoideum* is present, Pf2 is now able to outcompete Pf3 when grown together by using the siderophores produced by Pf3 (*t* = 13.36, *p* < 0.001), in this case displaying completely differing competitive abilities compared to when they are grown separately. Competition between Pf2 and Pf3 under these conditions is presumably dominated the social interactions of pyochelin use. Although Pf3 has an advantage when grown separately as it over-expresses the iron chelating pyochelin, which is advantageous during iron limitation (**Figure 2D** and **Table 1**), in direct competition, our results seem to indicate that Pf2 is able to utilize the excess pyochelin produced by Pf2, acting like a social cheater. This interaction is particularly interesting because previous studies have identified *gac* mutants that act as social cheaters (the opposite of our findings, although other studies have also shown *gac* mutants to be adaptive (Driscoll et al., 2011), benefitting from a variety of cooperative behaviors without paying any associated costs (Jousset et al., 2009).

Somewhat more puzzling are our results when Pf2 and Pf3 are grown together under iron limitation and in the presence of *D. discoideum*. Both these environmental variables should favor Pf2, as it should be able to utilize the over expressed siderophores produced by Pf3, which in turn should be eaten by *D. discoideum*. Instead we see the opposite results of our predictions, with Pf3 outcompeting Pf2 (*t* = 4.88, *p* < 0.001) (**Table 3**), suggesting that these environmental variables (i.e., the presence of *D. discoideum* and absence of iron) that are individually beneficial to Pf2, reduce each other’s effects in an predator-by-environment (G × E × E) interaction (Thompson, 1999; Wade, 2007). The biological rationale for this interaction is unclear. However, it is interesting to note that *D. discoideum*, which performs relatively poorly under iron limitation, grows better in the presence of Pf2 (**Figure 3B**). Previous work has identified that Pf2 produces chromene, a compound that actively increases *D. discoideum* growth (Stallforth et al., 2013), but it could be equally plausible that expression changes in Pf2 under iron limitation render it edible thereby increasing *D. discoideum* growth and reducing it’s competitive advantage compared to Pf3.

## Discussion

Competition between these two different bacteria (Pf2 and Pf3/Pf2 Δ*gacA*) then, largely seems to rely on the environmental conditions they experience. Neither the presence of iron nor *D. discoideum* could alone predict the competitive success of these competing bacteria, and the interactions of these two environmental conditions created unexpected outcomes. When grown separately, Pf2 reached higher densities (than Pf3 and Pf2Δ*gacA*) only in one treatment condition (iron-rich media and in the presence of *D. discoideum*, **Figure 2A**). However, when Pf2 is in direct competition with Pf3, it outcompetes it in two of the four treatments (**Figures 2A,D**), suggesting that these strains may only be able to coexist in fluctuating environments.

It is also important to note that we have only tested the competitive abilities of Pf2 and Pf3 under relatively simple environmental conditions and more complex, natural environments may allow for stable coexistence (or at least less drastic differences in competitive fitness). However, these conditions (iron limitation and *D. discoideum*) are likely to be extremely important in both the ecology and evolution for both strains. Both strains were isolated together from *D. discoideum*, and only one of them (Pf3) is edible. Furthermore, during the characterization of these strains, one of the largest differences in the secreted chemical profiles was the drastically increased expression of pyochelin, important for iron acquisition in iron-limited environments (Stallforth et al., 2013).

We have also not considered other ecological factors such as negative frequency dependent selection and snowdrift dynamics, which may play an important role in determining the outcome of this competition (Ross-Gillespie et al., 2007; Gore et al., 2009). In the case of public goods production, it has previously been shown that siderophore cheaters perform better when rare in the population (Ross-Gillespie et al., 2007). However, negative frequency dependent selection through predation may be equally important for Pf3: It may suffer the effects of predation more when common (Allen, 1988). Similarly, density dependent effects on both *D. discoideum* and the *P. fluorescens* strains can also have important consequences for this competition. For example, siderophore cheaters perform better under high population densities (Ross-Gillespie et al., 2009). Taken together these frequency and density dependent interactions might result in interesting and potentially complex feedback loops, which allow both strains to stably coexist.

These fluctuations in bacterial fitness, which depend on *D. discoideum* and iron, may provide some insight into the conditions that led to the evolution Pf3 from the ancestral Pf2 strain (Stallforth et al., 2013). In particular, it is difficult to explain why a mutation that renders a Pf3 edible should persist. However, our results suggest that the fitness effects of Pf3’s edibility depend on environmental context, and mutations in *gacA* may provide other benefits such as an increased growth rate irrespective of environmental conditions. Moreover, iron is likely to be relatively important nutrient for both *P. fluorescens* strains, with its patchy distribution within soil and low bioavailability (Kraemer, 2004), favoring adaptions that increase iron acquisition such as the up-regulation of pyochelin production (Dumas et al., 2013). However, it is important to note that there may also be some benefit to edibility because *D. discoideum* is able to disperse bacteria to new habitats, providing bacteria with new sources of food (Brock et al., 2011; DiSalvo et al., 2015). This suggests that differences between Pf2 and Pf3 may in part be due to transition from a specialized and mutualistic association between Pf2 and *D. discoideum* to a free-living environmental generalist in Pf3.

More generally our results suggest that making predictions about the microbial composition and competitive ability, even in the extremely simple microbiome of *D. discoideum*, is challenging not just because of species interactions but also because of predator-by-environment (G × E × E) interactions (Thompson, 1999; Wade, 2007). Nevertheless, with the increasing interest in understanding how complex mammalian microbiomes respond to changing environments and medical interventions, using ecological and evolutionary theory to test how simple microbiomes respond may offer the best hope for beginning to understand these kinds of inter-species interactions.

## Author Contributions

RI conceived the study, performed experiments, analyzed the data, and wrote the paper. OA performed experiments and wrote the paper. ER performed experiments and wrote the paper. DQ conceived the study, analyzed the data, and wrote the paper. JS conceived the study, analyzed the data, and wrote the paper.

## Conflict of Interest Statement

The authors declare that the research was conducted in the absence of any commercial or financial relationships that could be construed as a potential conflict of interest.

## References

[B1] Allen J. A. (1988). Frequency-dependent selection by predators. *Philos. Trans. R. Soc. Lond. B Biol. Sci.* 319 485–503. 10.1098/rstb.1988.0061 2905488

[B2] Biernaskie J. M. Gardner A. West S. A. (2013). Multicoloured greenbeards, bacteriocin diversity and the rock-paper-scissors game. *J. Evol. Biol.* 26 2081–2094. 10.1111/jeb.12222 23980628

[B3] Brock D. A. Callison W. E. Strassmann J. E. Queller D. C. (2016a). Sentinel cells, symbiotic bacteria and toxin resistance in the social amoeba *Dictyostelium discoideum*. *Proc. Biol. Sci.* 283:20152727. 10.1098/rspb.2015.2727 27097923 PMC4855374

[B4] Brock D. A. Jones K. Queller D. C. Strassmann J. E. (2016b). Which phenotypic traits of *Dictyostelium* discoideum farmers are conferred by their bacterial symbionts? *Symbiosis* 68 39–48. 10.1007/s13199-015-0352-0

[B5] Brock D. A. Douglas T. E. Queller D. C. Strassmann J. E. (2011). Primitive agriculture in a social amoeba. *Nature* 469 393–396. 10.1038/nature09668 21248849

[B6] Brock D. A. Read S. Bozhchenko A. Queller D. C. Strassmann J. E. (2013). Social amoeba farmers carry defensive symbionts to protect and privatize their crops. *Nat. Commun.* 4:2385. 10.1038/ncomms3385 24029835

[B7] Choi K. H. Schweizer H. P. (2006). mini-Tn7 insertion in bacteria with single *attTn7* sites: example *Pseudomonas aeruginosa*. *Nat. Protoc.* 1 153–161. 10.1038/nprot.2006.24 17406227

[B8] Cordero O. X. Ventouras L. A. DeLong E. F. Polz M. F. (2012a). Public good dynamics drive evolution of iron acquisition strategies in natural bacterioplankton populations. *Proc. Natl. Acad. Sci. U.S.A.* 109 20059–20064. 10.1073/pnas.1213344109 23169633 PMC3523850

[B9] Cordero O. X. Wildschutte H. Kirkup B. Proehl S. Ngo L. Hussain F. (2012b). Ecological populations of bacteria act as socially cohesive units of antibiotic production and resistance. *Science* 337 1228–1231. 10.1126/science.1219385 22955834

[B10] Coyte K. Z. Schluter J. Foster K. R. (2015). The ecology of the microbiome: networks, competition, and stability. *Science* 350 663–666. 10.1126/science.aad2602 26542567

[B11] DiSalvo S. Haselkorn T. S. Bashir U. Jimenez D. Brock D. A. Queller D. C. (2015). *Burkholderia* bacteria infectiously induce the proto-farming symbiosis of *Dictyostelium* amoebae and food bacteria. *Proc. Natl. Acad. Sci. U.S.A.* 112 E5029–E5037. 10.1073/pnas.1511878112 26305954 PMC4568666

[B12] Driscoll W. W. Pepper J. W. Pierson L. S. III Pierson E. A. (2011). Spontaneous Gac mutants of *Pseudomonas* biological control strains: cheaters or mutualists? *Appl. Environ. Microbiol.* 77 7227–7235. 10.1128/AEM.00679-11 21873476 PMC3194857

[B13] Dumas Z. Ross-Gillespie A. Kummerli R. (2013). Switching between apparently redundant iron-uptake mechanisms benefits bacteria in changeable environments. *Proc. Biol. Sci.* 280:20131055. 10.1098/rspb.2013.1055 23760867 PMC3712426

[B14] Friman V. P. Diggle S. P. Buckling A. (2013). Protist predation can favour cooperation within bacterial species. *Biol. Lett.* 9:20130548. 10.1098/rsbl.2013.0548 23945212 PMC3971697

[B15] Gentleman R. C. Carey V. J. Bates D. M. Bolstad B. Dettling M. Dudoit S. (2004). Bioconductor: open software development for computational biology and bioinformatics. *Genome. Biol.* 5:R80. 15461798 10.1186/gb-2004-5-10-r80 PMC545600

[B16] Gore J. Youk H. van Oudenaarden A. (2009). Snowdrift game dynamics and facultative cheating in yeast. *Nature* 459 253–256. 10.1038/nature07921 19349960 PMC2888597

[B17] Griffin A. S. West S. A. Buckling A. (2004). Cooperation and competition in pathogenic bacteria. *Nature* 430 1024–1027. 10.1038/nature02744 15329720

[B18] Hassan K. A. Johnson A. Shaffer B. T. Ren Q. Kidarsa T. A. Elbourne L. D. (2010). Inactivation of the GacA response regulator in *Pseudomonas* fluorescens Pf-5 has far-reaching transcriptomic consequences. *Environ. Microbiol.* 12 899–915. 10.1111/j.1462-2920.2009.02134.x 20089046

[B19] Inglis R. F. Biernaskie J. M. Gardner A. Kummerli R. (2016). Presence of a loner strain maintains cooperation and diversity in well-mixed bacterial communities. *Proc. Biol. Sci.* 283:20152682. 10.1098/rspb.2015.2682 26763707 PMC4721107

[B20] Ishii Y. Shimada M. (2012). Learning predator promotes coexistence of prey species in host-parasitoid systems. *Proc. Natl. Acad. Sci. U.S.A.* 109 5116–5120. 10.1073/pnas.1115133109 22411808 PMC3324012

[B21] Jousset A. Rochat L. Pechy-Tarr M. Keel C. Scheu S. Bonkowski M. (2009). Predators promote defence of rhizosphere bacterial populations by selective feeding on non-toxic cheaters. *ISME J.* 3 666–674. 10.1038/ismej.2009.26 19322247

[B22] Jousset A. Rochat L. Scheu S. Bonkowski M. Keel C. (2010). Predator-prey chemical warfare determines the expression of biocontrol genes by rhizosphere-associated *Pseudomonas* fluorescens. *Appl. Environ. Microbiol.* 76 5263–5268. 10.1128/AEM.02941-09 20525866 PMC2916451

[B23] Kerr B. Riley M. A. Feldman M. W. Bohannan B. J. M. (2002). Local dispersal promotes biodiversity in a real-life game of rock-paper-scissors. *Nature* 418 171–174. 10.1038/nature00823 12110887

[B24] Kirkup B. C. Riley M. A. (2004). Antibiotic-mediated antagonism leads to a bacterial game of rock-paper-scissors *in vivo*. *Nature* 428 412–414. 10.1038/nature02429 15042087

[B25] Kraemer S. M. (2004). Iron oxide dissolution and solubility in the presence of siderophores. *Aquat. Sci.* 66 3–18. 10.1007/s00027-003-0690-5

[B26] Kummerli R. Brown S. P. (2010). Molecular and regulatory properties of a public good shape the evolution of cooperation. *Proc. Natl. Acad. Sci. U.S.A.* 107 18921–18926. 10.1073/pnas.1011154107 20944065 PMC2973908

[B27] Leinweber A. Fredrik Inglis R. Kummerli R. (2017). Cheating fosters species co-existence in well-mixed bacterial communities. *ISME J.* 11 1179–1188. 10.1038/ismej.2016.195 28060362 PMC5437929

[B28] Meyer J. R. Kassen R. (2007). The effects of competition and predation on diversification in a model adaptive radiation. *Nature* 446 432–435. 10.1038/nature05599 17377581

[B29] R Core Team (2015). *R: A Language and Environment for Statistical Computing*. Vienna: R Foundation for Statistical Computing Available at: https://www.r-project.org/

[B30] Ross-Gillespie A. Gardner A. Buckling A. West S. A. Griffin A. S. (2009). Density dependence and cooperation: theory and a test with bacteria. *Evolution Int. J. Org. Evolution* 63 2315–2325. 10.1111/j.1558-5646.2009.00723.x 19453724

[B31] Ross-Gillespie A. Gardner A. West S. A. Griffin A. S. (2007). Frequency dependence and cooperation: theory and a test with bacteria. *Am. Nat.* 170 331–342. 10.1086/519860 17879185

[B32] Schwyn B. Neilands J. B. (1987). Universal chemical assay for the detection and determination of siderophores. *Anal. Biochem.* 160 47–56. 10.1016/0003-2697(87)90612-9 2952030

[B33] Smith J. Strassmann J. E. Queller D. C. (2016). Fine-scale spatial ecology drives kin selection relatedness among cooperating amoebae. *Evolution* 70 848–859. 10.1111/evo.12895 26931797

[B34] Stallforth P. Brock D. A. Cantley A. M. Tian X. Queller D. C. Strassmann J. E. (2013). A bacterial symbiont is converted from an inedible producer of beneficial molecules into food by a single mutation in the *gacA* gene. *Proc. Natl. Acad. Sci. U.S.A.* 110 14528–14533. 10.1073/pnas.1308199110 23898207 PMC3767522

[B35] Steiner U. K. (2007). Investment in defense and cost of predator-induced defense along a resource gradient. *Oecologia* 152 201–210. 10.1007/s00442-006-0645-3 17221255

[B36] Thompson J. N. (1999). Specific hypotheses on the geographic mosaic of coevolution. *Am. Nat.* 153 S1–S14. 10.1086/303208

[B37] van den Broek D. Bloemberg G. V. Lugtenberg B. (2005). The role of phenotypic variation in rhizosphere *Pseudomonas* bacteria. *Environ. Microbiol.* 7 1686–1697. 10.1111/j.1462-2920.2005.00912.x 16232284

[B38] Wade M. J. (2007). The co-evolutionary genetics of ecological communities. *Nat. Rev. Genet.* 8 185–195. 10.1038/nrg2031 17279094

