# Supplementary material for: Predator-by-Environment Interactions Mediate Bacterial Competition in the Dictyostelium discoideum Microbiome
Source: Front Microbiol. 2018 Apr 24;9:781. doi: 10.3389/fmicb.2018.00781 (PMC5928206; doi:10.3389/fmicb.2018.00781)
Supplement: Supplementary file 1 [file Data_Sheet_1.DOCX]

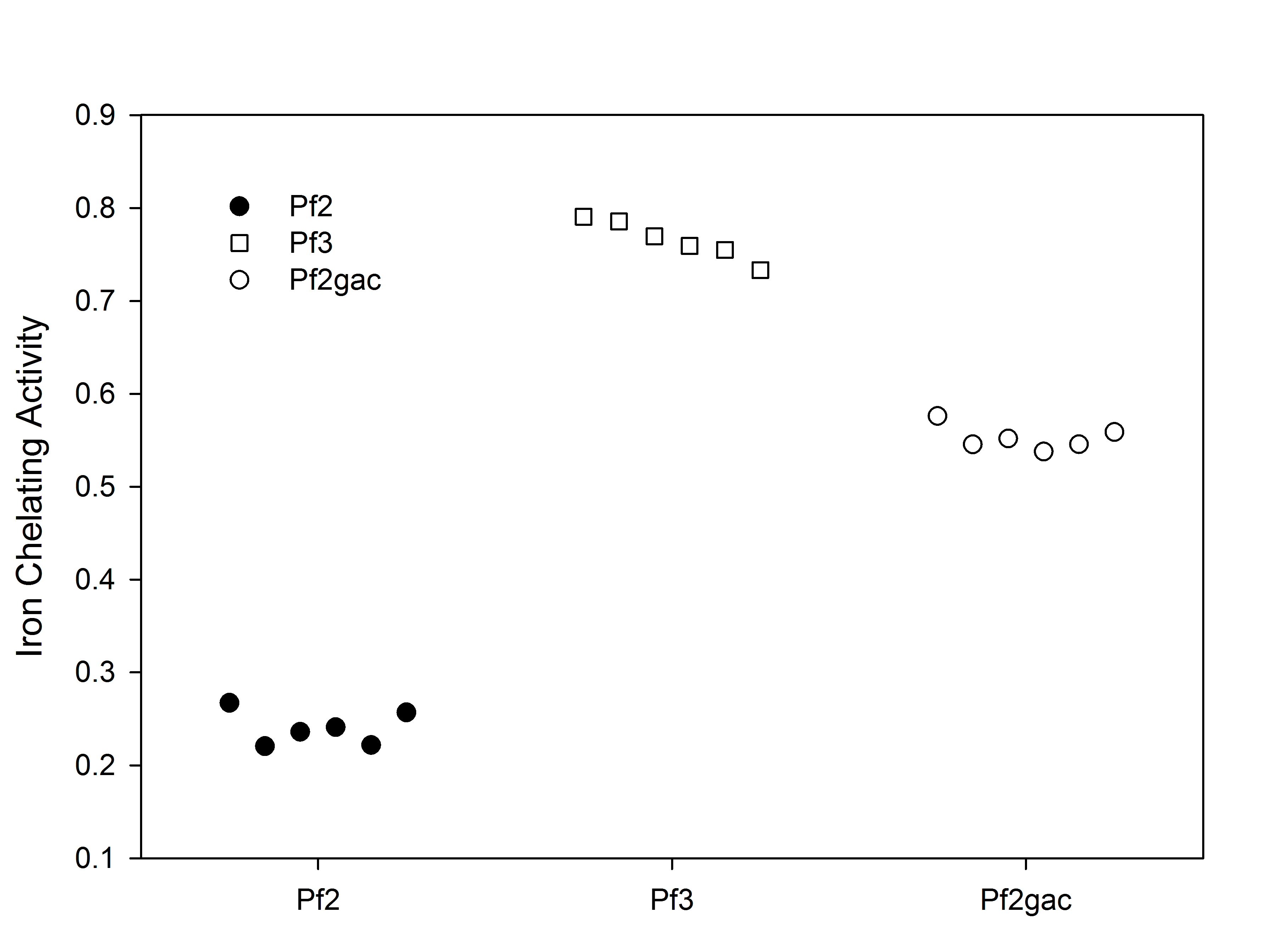


Figure S1. A CAS-assay was performed to measure differences in iron-chelating ability between *D. discoideum* associated strains. Iron chelation varied significantly between strains (*F_2,15_* = 1281 , p < 0.001) with both Pf3 and Pf2Δ*gacA* displayed higher levels of iron chelation compared to Pf2 (Tukey HSD, p < 0.001). Pf3 also displayed significantly higher levels of iron chelation when compared to Pf2Δ*gacA* (Tukey HSD, p < 0.001). This suggests that Pf3 and Pf2Δ*gacA* should be able to grow better under iron limitation.


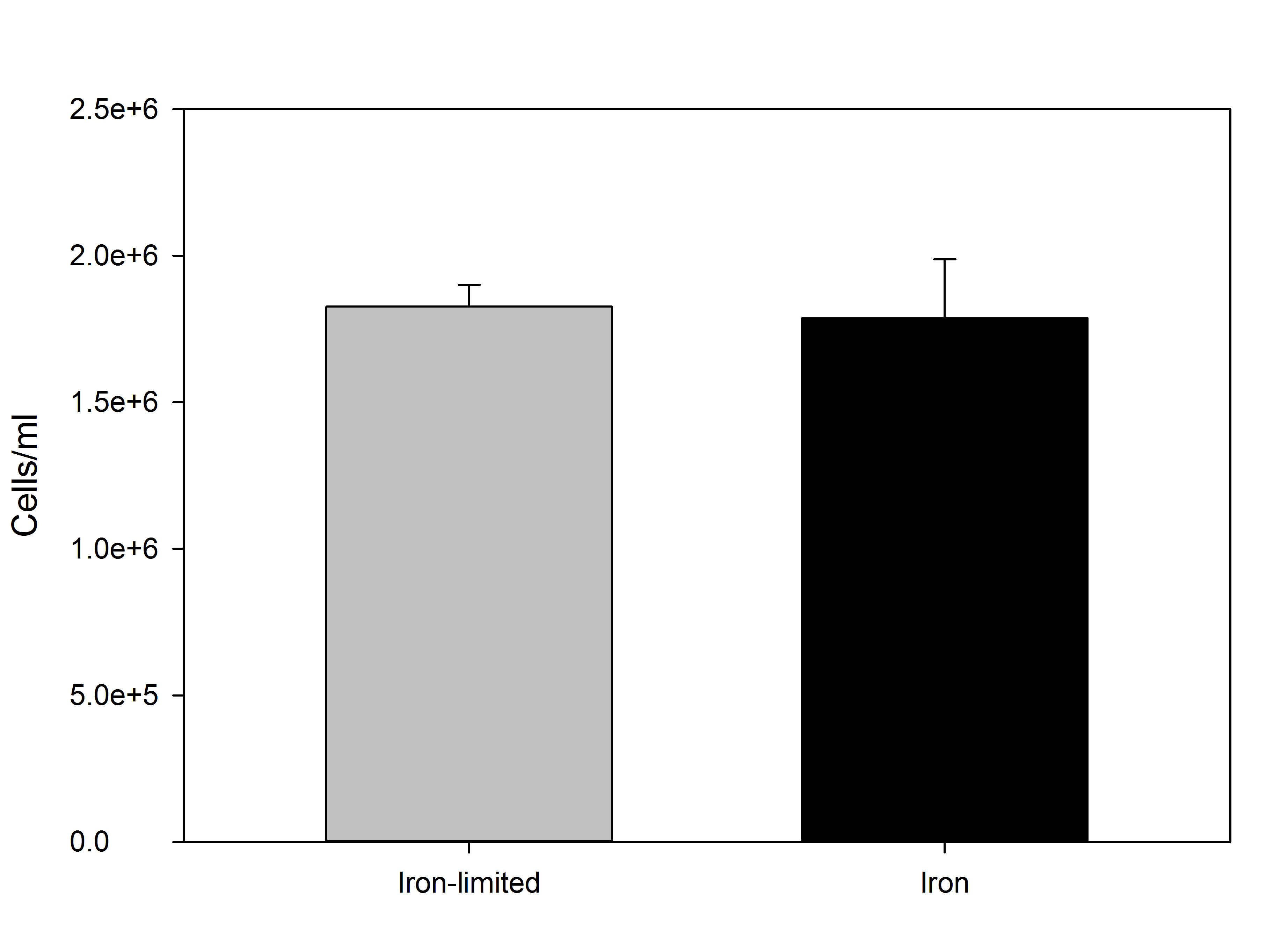


Figure S2. *D. discoideum* densities when grown in iron-limited and iron-rich environments. Amoebae were grown on CAA agar supplemented with either 100uM FeCl_3_ (for relatively iron rich environments) or with 20mM NaHCO_3_ (sodium bicarbonate) and 100 μg/ml human apo-transferrin (for relatively iron poor environments), 3 replicates for each treatment. Heat killed *K. pneumoniae*, created by exposing bacteria in liquid culture to 80C for 30 minutes, were provided as a food source for the amoebae. No difference was observed between treatments (*t* = 0.186, *p* > 0.86).
